# Achieving clinical outcomes with benralizumab in severe eosinophilic asthma patients in a real-world setting: ORBE II study

**DOI:** 10.1186/s12931-023-02539-7

**Published:** 2023-09-28

**Authors:** Alicia Padilla-Galo, Isabel Moya Carmona, Pilar Ausín, Luis Carazo Fernández, Ismael García-Moguel, José Luis Velasco-Garrido, Rubén Andújar-Espinosa, Francisco Casas-Maldonado, Eva Martínez-Moragón, Carlos Martínez Rivera, Elisabet Vera Solsona, Fernando Sánchez-Toril López, Andrea Trisán Alonso, Marina Blanco Aparicio, Marcela Valverde-Monge, Borja Valencia Azcona, Marta Palop Cervera, Javier Nuevo, Jesús Sánchez Tena, Gustavo Resler, Elisa Luzón, Alberto Levy Naon

**Affiliations:** 1 Hospital Costa del Sol, Autovía A-7 Km 187, Marbella, Málaga, 29603 Spain; 2 H. Virgen de la Victoria, Málaga, Spain; 3 H. del Mar, Universidad Pompeu Fabra University (UPF), Barcelona, Spain; 4 Complejo Asistencial Universitario de León, León, Spain; 5 H. U. 12 de Octubre, Instituto de Investigación Sanitaria Hospital 12 de Octubre (Imas12), Madrid, Spain; 6 H. U. Virgen de la Victoria, Málaga, Spain; 7 H. U. Virgen de la Arrixaca, Murcia, Spain; 8 H. U. Clínico San Cecilio, Granada, Spain; 9 H. U. Doctor Peset, Valencia, Spain; 10 H. U. Germans Trias i Pujol, Barcelona, Spain; 11 H. U. Miguel Servet, Zaragoza, Spain; 12 H. Arnau de Vilanova-Lliria, Valencia, Spain; 13 H. U. Puerta de Hierro Majadahonda, Madrid, Spain; 14 H. U. A Coruña, A Coruña, Spain; 15 H. U. Fundación Jiménez Diaz, Madrid, Spain; 16 H. de Sagunto, Valencia, Spain; 17 Medical Department, AstraZeneca Farmacéutica S.A, Madrid, Spain; 18 H. Quirón Salud, Málaga, Spain

**Keywords:** Benralizumab, Severe eosinophilic asthma, ORBE II, Real world evidence, Biologics, Chronic rhinosinusitis with nasal polyps, Asthma exacerbations, Spain

## Abstract

**Background:**

The ORBE II study aimed to describe the characteristics and clinical outcomes of adult patients with severe eosinophilic asthma (SEA) treated with benralizumab in a real-world setting in Spain.

**Methods:**

ORBE II (NCT04648839) was an observational, retrospective cohort study in adult SEA patients who had been prescribed benralizumab. Demographic and clinical data of 204 SEA patients were collected 12 months prior to benralizumab initiation (baseline) and at follow-up. Exacerbation rate, asthma symptoms, maintenance oral corticosteroid (OCS) use and lung function were evaluated, among other variables.

**Results:**

A total of 204 SEA patients were evaluated. Mean (standard deviation, SD) age of the study population was 56.4 (12.4) years, 62.3% were women and mean (SD) duration of asthma was 15.1 (12.7) years. Median (Q1–Q3) follow-up duration was 19.5 (14.2–24.2) months. At baseline, 72.6% of the overall population (OP) presented blood eosinophil counts ≥ 300 cells/µL; 36.8% had comorbid chronic rhinosinusitis with nasal polyps (CRSwNP); 84.8% reported at least one severe exacerbation, and 29.1% were OCS-dependent. At 1 year of follow-up, patients receiving benralizumab treatment had a 85.6% mean reduction in exacerbations from baseline, and 81.4% of patients achieved zero exacerbations. We also found a clinically relevant mean (SD) increase in pre-bronchodilator (BD) FEV_1_ of 331 (413) mL, with 66.7% of patients achieving a pre-BD FEV_1_ increase ≥ 100 mL, and 46.3% of patients achieving a pre-BD FEV_1_ ≥ 80% of predicted. Regarding symptom control, 73.8% of the OP obtained an ACT score ≥ 20 points. After 1 year of follow-up, mean reduction in the daily OCS dose was 70.5%, and complete OCS withdrawal was achieved by 52.8% of the OCS-dependent patients. Almost half (43.7%) of the OP on benralizumab met all four criteria for clinical remission. Patients with concomitant CRSwNP obtained similar or enhanced outcomes.

**Conclusions:**

These data support the real-world benefits of benralizumab in SEA patients, and particularly in those with concomitant CRSwNP.

**Trial registration:**

NCT04648839.

**Supplementary Information:**

The online version contains supplementary material available at 10.1186/s12931-023-02539-7.

## Background

Severe asthma (SA) is defined as asthma that requires the use of multiple drugs to achieve control, including high-dose inhaled corticosteroids (ICS) combined with other controller medications such as long-acting β2-adrenergic agonists (LABA) with or without oral corticosteroids (OCS). However, it may remain uncontrolled despite the use of this high intensity therapeutic approach [1–4]. SA is associated with more frequent exacerbations than non-severe asthma, as well as increased use of healthcare resources [1, 5, 6]. Asthma clinical guidelines and recommendations highlight the importance of a personalized therapeutic strategy aimed at achieving clinical improvements in several domains, including early and sustained control of symptoms, prevention or minimisation of both asthma exacerbations and lung function decline, tapering of OCS (in order to avoid OCS-related adverse effects) and a reduction in asthma-associated mortality [3, 7].

The most common SA phenotype is type 2 (T2) severe eosinophilic asthma (SEA), which is characterised by the presence of eosinophils in blood and sputum, as well as by clinical features such as comorbid chronic rhinosinusitis with nasal polyps (CRSwNP) [8]. SEA patients experience frequent exacerbations and often rely on high-dose OCS, which are strongly associated with major adverse events, in order to manage or prevent their occurrence [7]. Exacerbations are also associated with reduced pulmonary function and a significant increase in hospitalizations [5, 9].

The addition of biologics to the therapeutic armamentarium has helped to address all the clinical challenges associated with SEA, such as reducing exacerbations and/or the burden of OCS as a maintenance or sporadic treatment [1]. Given the ability of biologics to reduce SA clinical burden, the achievement of clinical remission has recently been proposed as a goal of asthma management. Different expert consensuses have suggested different definitions for a super-response to biologics and clinical remission in SA [10, 11].

One of the currently available biologics approved for the treatment of SEA is benralizumab, an anti-eosinophil monoclonal antibody that binds to the alpha subunit of the interleukin-5 receptor (IL-5Rα) expressed by cells such as eosinophils and basophils, inducing their apoptosis through an antibody-dependent cell-mediated cytotoxicity mechanism [12]. The efficacy and safety of benralizumab in SA were demonstrated in three pivotal phase 3 trials: SIROCCO (ClinicalTrials.gov NCT01928771) [13], CALIMA (ClinicalTrials.gov NCT01914757) [14], and ZONDA (ClinicalTrials.gov NCT02075255) [15], which included nearly 3000 patients. Based on all the findings that confirmed a reduction in asthma exacerbations, improved lung function and asthma control, and reduction or elimination of the use of OCS, benralizumab obtained its marketing authorization in Europe in 2018 and was indicated as an adjunctive maintenance therapy for the management of adult patients with uncontrolled SEA. Integrated analyses of results from the pivotal and extension studies for up to 5 years of follow-up confirmed the long-term safety and efficacy of benralizumab [16–19]. The open-label, phase 3 PONENTE trial (ClinicalTrials.gov NCT03557307) showed that SEA patients treated with benralizumab could significantly reduce the use of OCS and even achieve complete OCS withdrawal, along with sustained or improved asthma control and a reduction in exacerbations using a personalised steroid-sparing strategy and an adrenal function assessment scheme that have been included in recent consensuses and guidelines [7, 20].

SA may be associated with CRSwNP in up to 40-45% of cases [21–24]. Asthma patients with concomitant CRSwNP often have increased airway obstruction, reduced asthma control, higher blood eosinophil and fraction of exhaled nitric oxide (FeNO) levels, a significantly higher number of asthma exacerbations per year, and greater reliance on OCS. Several studies, such as the phase 3 ANDHI trial (ClinicalTrials.gov, NCT03170271) have identified the presence of nasal polyposis (NP) in SA patients as a predictor of response to benralizumab and showed enhanced effects related to benralizumab in patients with concomitant CRSwNP [25–27].

The aim of the retrospective ORBE II study (as part of the XALOC programme, which includes more than 1500 patients from across nine countries and aims to assess the real-world effectiveness of benralizumab) was to characterise the profile of SEA patients, including the subset of patients with concomitant CRSwNP treated with benralizumab in real-life conditions in Spain and to analyse the benefits of benralizumab in several SEA domains. In addition, we present a post-hoc analysis of response and clinical remission.

## Methods

### Study design

ORBE II (ClinicalTrials.gov NCT04648839) was a multicentre, observational, retrospective cohort study of adult patients (≥ 18 years) diagnosed with SEA who were prescribed benralizumab by asthma specialists (pulmonologist/allergist) as per routine clinical practice after it was granted marketing authorization in Spain (January 2019). Patients fulfilled the currently approved European indication for benralizumab as an add-on therapy for adult patients with severe eosinophilic uncontrolled asthma despite being treated with high-dosage inhaled corticosteroids and long-acting β agonists. Patients who received benralizumab in a clinical trial during the observation period were not included. The primary objective was to characterise the profile and treatment patterns of SEA patients requiring benralizumab treatment. For this objective, patients who received at least one dose of benralizumab according to clinical practice were considered. The secondary objective was to evaluate clinical outcomes in all patients included who received at least three doses of subcutaneous benralizumab (30 mg every 4 weeks for the first three doses, and every 8 weeks thereafter) during follow-up.

Data were obtained retrospectively from the electronic medical records of the 15 participating asthma units. The index date was defined as the date of the first dose of benralizumab, which had to have occurred in the period between 1 and 2019 and 3 months before the specific study site initiation. The end of the follow-up period was up to enrolment of the patient in the study, allowing for a minimum of 3 months of follow-up. In this paper, we present the results corresponding to the first 12 months of follow-up.

Baseline sociodemographic and clinical data corresponded to the closest measure to index date, within the previous 12-month period. Data collected at baseline included age, sex, smoking habits, height, weight, body mass index (BMI), biomarkers (eosinophil counts in peripheral blood, total IgE levels and FeNO), asthma medications (including previous biological treatments) and asthma comorbidities (including CRSwNP).

Data on lung function (including forced expiratory volume in 1 s [FEV_1_], predicted FEV_1_%, forced vital capacity [FVC], and FEV_1_/FVC), asthma control (Asthma Control Test [ACT] [28, 29]), use of systemic/oral corticosteroids (CS) and exacerbations (referred to severe exacerbations, i.e., those requiring the use of systemic CS or the increase of the maintenance dose of OCS for at least three days or, hospitalization or emergency room visits due to asthma requiring the use of systemic CS) were collected to characterise the patients’ clinical outcomes in the year prior to and after benralizumab initiation. For the purpose of this analysis, corticosteroid-dependent patients were defined as those who received maintenance systemic CS treatments for at least 3 months within the 12 months prior to the index date. All the above-listed clinical variables were assessed for the overall population (OP) during follow-up and were also evaluated in the subgroups of patients with and without concomitant CRSwNP.

Additional analyses were performed to evaluate the degree of response to benralizumab based on previously published definitions. Super-responders to benralizumab have been commonly defined as those patients achieving complete exacerbation elimination and cessation of maintenance OCS use [11, 30]. Clinical remission is a more recent concept in SA and has been defined as the absence of exacerbations for 12 months, absence of significant symptoms, a relevant improvement in lung function, and no use of OCS for at least 12 months [10]. Based on these definitions, the following four pre-defined key criteria were used in our analyses to identify patients achieving a super-response to benralizumab and asthma clinical remission: no exacerbations; pre-bronchodilator (BD) FEV_1_ increase ≥ 100 mL; ACT score ≥ 20; and no maintenance OCS use.

### Statistical analysis

A descriptive statistical analysis was performed. General descriptive statistics for continuous numerical variables included the number of observations, mean, standard deviation (SD), and additionally, the median and quartiles 1 and 3 (Q1-Q3) when considered appropriate. For categorical variables, the frequency distribution and percentage of subjects with a certain event/characteristic was presented.

Missing values were not considered for calculating percentages or any other descriptive estimator, meaning that only valid values are presented. No use of any method for the handling of missing data was anticipated.

In accordance with the original study design, the results shown are purely descriptive. No p-values are presented (i.e., no formal hypothesis testing or multivariate analysis has been performed) since neither the study design nor the corresponding sample size estimation allow for this type of analysis.

The analysis was performed using the IBM SPSS Statistics software, Version 26.0 (IBM Corp. Armonk, NY).

## Results

### Baseline characteristics of the study population

A total of 204 patients recruited from 15 hospitals were analysed. Demographic and clinical data were collected at baseline. In summary, the mean (SD) age of the cohort was 56.4 (12.4) years, 62.3% were women, and the mean (SD) age at asthma onset was 34.4 (16.4) years. Most patients (93.9%) had comorbidities, with CRSwNP being the most frequent (36.8% of patients) (Table 1). A high proportion of the study population (72.6% of patients) presented baseline blood eosinophil counts ≥ 300 cells/µL and 38.3% presented FeNO levels ≥ 50 parts per billion (ppb) (Table 2). Based on data collected on previous treatments, 69.1% of the OP were biologic-naïve, while 30.9% of patients had received at least one previous biological treatment for SA.

**Table 1:** Baseline demographic characteristics of the study population

Parameters	Total N = 204
**Age (years), mean (SD)**	56.4 (12.4)
**Women, n/N (%)**	127/204 (62.3%)
**BMI (Kg/m** ^**2**^ **), mean (SD)** ^**a**^	28.1 (6.3)
*Obese (BMI ≥ 30 Kg/m*^*2*^*), n/N (%)*	54/188 (28.7%)
**Age at asthma onset (years), mean (SD)**	34.4 (16.4)
**Asthma duration (years), mean (SD)**	15.1 (12.7)
**Allergic asthma, n/N (%)** ^**b**^	68/204 (33.3%)
**Smoking history, n/N (%)** ^**c**^	
*Non-smoker*	128/203 (63.1%)
*Former smoker*	69/203 (34%)
*Smoker*	6/203 (3%)
**Comorbidities, n/N (%)**	191/204 (93.6%)
*CRSwNP*	75/204 (36.8%)
*Gastroesophageal reflux*	42/204 (20.6%)
*Dyslipidaemia*	36/204 (17.6%)
*Osteoporosis*	32/204 (15.7%)
*Arterial hypertension*	32/204 (15.7%)
*Sleep apnoea syndrome*	26/204 (12.7%)
*Type II diabetes mellitus*	18/204 (8.8%)
*Depression*	17/204 (8.3%)
*COPD*	14/204 (6.9%)
*Anxiety*	13/204 (6.4%)
*Hypothyroidism*	12/204 (5.9%)
*Cataracts*	6/204 (2.9%)

Due to the nature of this real-world study, sample sizes vary due to missing/ unavailable/ not valid

^a^Data missing from 16 patients; ^b^As determined by the investigator; ^c^Data missing from 1 patient

All percentages were calculated over the total of patients with valid data

BMI, body mass index; COPD, chronic obstructive pulmonary disease; CRSwNP, chronic rhinosinusitis with nasal polyposis; SD, standard deviation

**Table 2:** Baseline clinical characteristics of the study population

Parameters	Baseline
**Eosinophil count in peripheral blood**	N = 197
Eosinophil count (cells/µL), median [Q1-Q3]	500 [220–750]
*Patients with eos ≥ 300 cells/µL, n/N (%)*	143/197 (72.6%)
*Patients with eos ≥ 500 cells/µL, n/N (%)*	100/197 (50.8%)
**Total serum IgE**	**N = 144**
*IgE level at index date (IU/mL), median [*Q1-Q3*]*	163 [49.8–432]
**FeNO**	**N = 120**
FeNO (ppb), median [Q1-Q3]	36.8 [19.2–64]
*FeNO < 25 ppb, n/N (%)*	44/120 (36.7%)
*FeNO ≥ 25 ppb < 50 ppb, n/N (%)*	30/120 (25.0%)
*FeNO ≥ 50 ppb, n/N (%)*	46/120 (38.3%)
**Patients with previous biological treatments, n/N (%)** ^**a**^	63/204 (30.9%)
*1 previous line*	57/204 (27.9%)
*2 previous lines*	5/204 (2.5%)
*3 previous lines*	1/204 (1.6%)
*Omalizumab*	32/203 (15.7%)
*Mepolizumab*	33/203 (16.3%)
*Reslizumab*	5/203 (2.5%)
**Other previous asthma treatments, n/N (%)**	
*LAMA*	152/204 (74.5%)
*LTRA*	117/204 (57.4%)
**Use of OCS**	**N = 182**
OCS-dependent patients, n/N (%)^**b**^	53/182 (29.1%)
*Daily OCS dose (mg), mean (SD)*	19.7 (15.8)
*Daily OCS dose (mg), median [*Q1-Q3*]* *Daily OCS dose > 5 mg/day, n/N (%)*	15 [6.9–29.0] 41/53 (77.4%)
**Severe exacerbations**	**N = 204**
Patients with severe exacerbations, n/N (%)	173/204 (84.8%)
Severe exacerbations, mean (SD)	2.5 (2.3)
**Lung function**	**N = 170**
Pre-BD FEV_1_ (mL), mean (SD)	1909 (780)^**c**^
Pre-BD FEV_1_ (% predicted), mean (SD)	67.3 (21.0)
Patients with pre-BD FEV_1_ ≥ 80%, n/N (%)	50/170 (29.4%)
**Asthma control**	**N = 148**
ACT score, mean (SD)	14.1 (5.1)
ACT score ≥ 20, n/N (%)	24/148 (16.2%)

“N” represents the total number of patients with valid data

^a^Patients could have received more than 1 biologic. A total of 141 patients (61.9%) had not received any previous line of biologics^; b^Corticosteroid-dependent patients were defined as those who received maintenance systemic corticosteroid treatments for at least 3 months within the 12 months prior to the index date.^; c^Missing/ unavailable/ invalid data from 16 patients

ACT, asthma control test; FeNO, fractional exhaled nitric oxide; FEV_1_, forced expiratory volume in the first second; IgE, immunoglobulin E; quartiles 1 and 3 (Q1-Q3); LAMA, long-acting muscarinic antagonists; LTRA, leukotriene receptor antagonists; OCS, oral corticosteroids; SD, standard deviation

In the year prior to starting benralizumab treatment, only 15.2% of patients were free of exacerbations. The mean (SD) number of severe exacerbations during the year prior to benralizumab was 2.5 (2.3) (Table 2). According to the pattern of use of systemic OCS, 29.1% of patients were OCS-dependent, with a median [Q1-Q3] daily dose of 15 [6.9–29] mg **(**Table 2). The OP presented poor control of asthma symptoms, with 83.8% of patients having an ACT score < 20 points and a mean (SD) ACT score of 14.1 (5.1) points at baseline. In terms of lung function, the mean (SD) pre-BD FEV_1_ was 67.3% (21%) of predicted while for 70.6% of patients, it was < 80% of predicted.

In the subset of biologic-naïve patients, we found a slightly higher baseline proportion of patients presenting eosinophil counts ≥ 300 cells/ µL (80.3%), severe exacerbations (87.2%) and poor asthma control (86.1% had an ACT score <20 points) (data not shown).

### Biomarker dynamics and clinical outcomes of the study population receiving benralizumab treatment

The median [Q1-Q3] follow-up of patients was 19.5 [14.2–24.2] months and the median [Q1-Q3] duration of benralizumab treatment was 18.3 [12.3–23.4] months. At 1 year of follow-up (1-year FUP), only 11 patients (5.4%) had discontinued benralizumab, mostly due to a suboptimal response (Additional file 1: Table S1). Data collected from patients receiving benralizumab confirmed an overall decrease in T2 inflammation biomarkers at 1-year FUP. Median [Q1-Q3] blood eosinophil counts fell from a median [Q1-Q3] of 500 [220–750] cells/µL at baseline to 0 [0–0] cells/µL at 1 year. Median [Q1-Q3] FeNO levels also decreased from 36.8 [19.2–64] ppb to 24.9 [15.9–55.3] ppb (Additional file 1: Table S2).

**Fig. 1:**
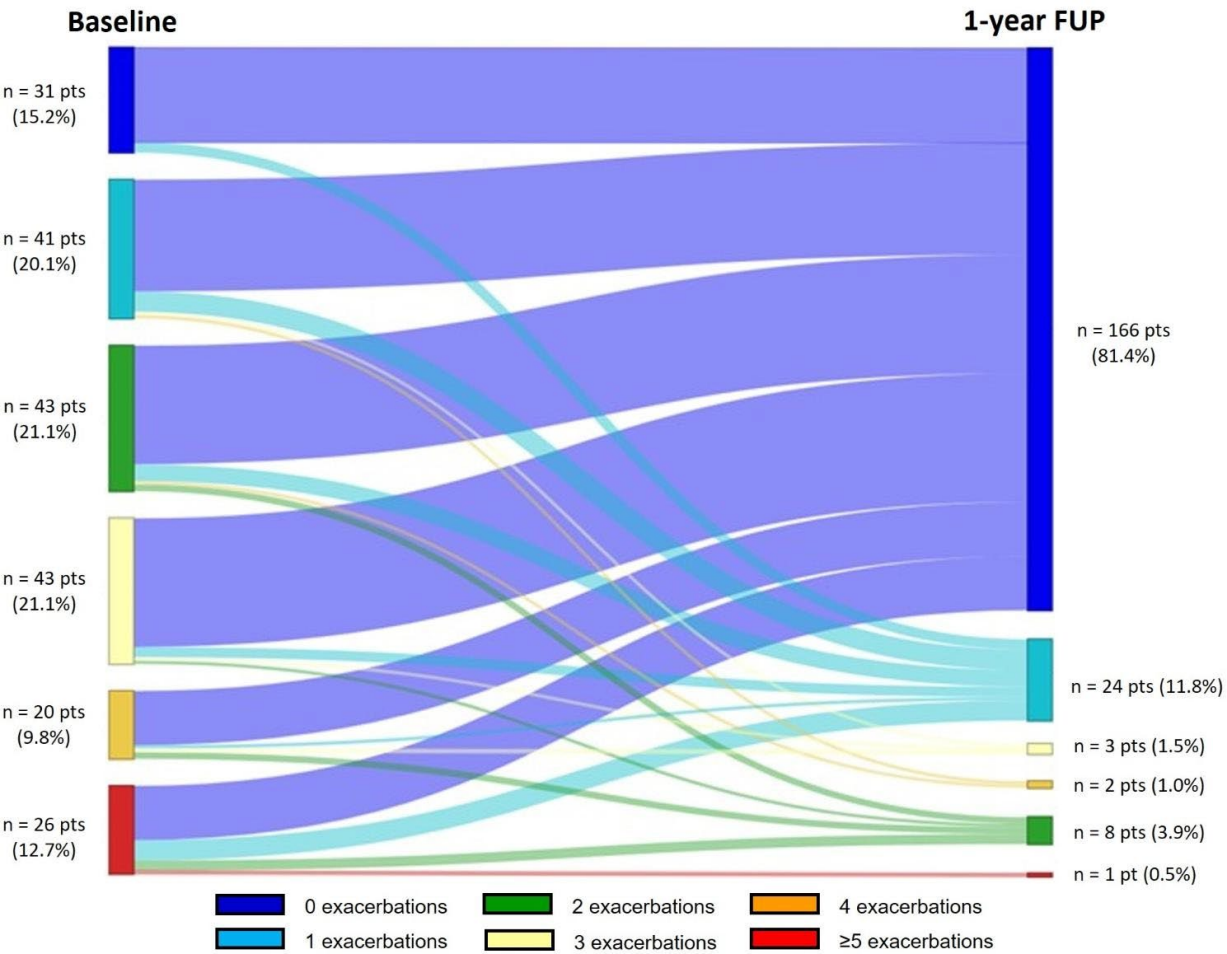
Change in the number of exacerbations over 1-year follow-up. The number of exacerbations per patient is presented in the Sankey diagram, which shows transition between the six exacerbation categories (from 0 exacerbations to ≥ 5 exacerbations) from baseline to 1 year of follow-up. The width at each time point is proportional to the number of patients in the category. FUP, follow-up; Pts, patients

As regards to clinical outcomes, there was an overall 85.6% reduction of exacerbations at 1-year FUP from baseline. Exacerbations fell from a mean (SD) of 2.5 (2.3) exacerbations during the year prior to benralizumab to 0.36 (0.98) at 1-year FUP (Table 3, Additional file 1: Figure S1A). We also found an increase in the proportion of patients with zero exacerbations, which increased from 15.2% at baseline to 81.4% at 1-year FUP (Fig. 1; Table 3**)**. Mean percentage reductions of 75% and 88.9% were observed in the number of hospitalizations and emergency department (ED) visits at 1-year FUP (Table 3).

**Table 3:** Clinical outcomes of the overall population receiving benralizumab treatment at 1-year of follow-up

Parameters	Baseline	1-year FUP
Severe exacerbations	N = 204	N = 204
Severe exacerbations, mean (SD)	2.5 (2.3)	0.36 (0.98)
Patients with zero severe exacerbations, n/N (%)	31/204 (15.2%)	166/204 (81.4%)
**Changes in severe exacerbations**		**N = 173**
Patients with reduction in exacerbations, n/N (%)		161/173 (93.1%)
Patients achieving ≥ 50% reduction in severe exacerbations, n/N (%)		159/173 (91.9%)
Percentage reduction in severe exacerbations		85.6%^**a**^
**Asthma-related healthcare resource use**	**N = 204**	** N = 204**
Patients with no hospitalizations, n/N (%)	159/203 (78.3%)	196/204 (96.1%)
Hospitalizations, mean (SD)	0.4 (0.9)	0.1 (0.3)
Patients with no ED visits, n/N (%)	118/204 (57.8%)	188/204 (92.2%)
ED visits, mean (SD)	0.9 (1.8)	0.1 (0.4)
**Changes in use of asthma-related healthcare resources**		**N = 204**
Percentage reduction in mean hospitalizations		75%
Percentage reduction in ED visits		88.9%^a^
**Lung function**	**N = 170**	** N = 134**
Pre-BD FEV_1_ (mL), mean (SD)	1909 (780)^b^	2186 (809)^c^
Pre-BD FEV_1_ (% predicted), mean (SD)	67.3 (21.0)	78.7 (22.2)
Patients with pre-BD FEV_1_ ≥ 80%, n/N (%)	50/170 (29.4%)	62/134 (46.3%)
**Changes in lung function**		**N = 114**
Pre-BD FEV_1_ increment ≥ 100mL, n/N (%)		76/114 (66.7)
Increase in pre-BD FEV_1_ (mL), mean (SD)		331 (413)
**Asthma control**	**N = 148**	** N = 130**
ACT score, mean (SD)	14.1 (5.1)	20.9 (4.8)
Patients with ACT score ≥ 20, n/N (%)	24/148 (16.2%)	96/130 (73.8%)
Patients with ACT score increase ≥ 3, n/N (%)		75/104 (72.1%)
Increase in ACT score, mean (SD)		6.6 (6.0)^**d**^

Due to the nature of this real-world study, sample sizes vary due to missing/unavailable/invalid data. “N” represents the total number of patients with valid data. All percentages were calculated over the total number of patients with valid data unless otherwise specified

^**a**^Percentage calculated out of a total of 204 patients; ^**d**^mean calculated over 104 patients; ^b^Data missing from 16 patients: ^c^Data missing from 7 patients

ACT, asthma control test; ED, emergency department; FEV_1_, forced expiratory volume in the first second; FUP, follow-up; pre-BD, pre-bronchodilator; SD, standard deviation

**Fig. 2:**
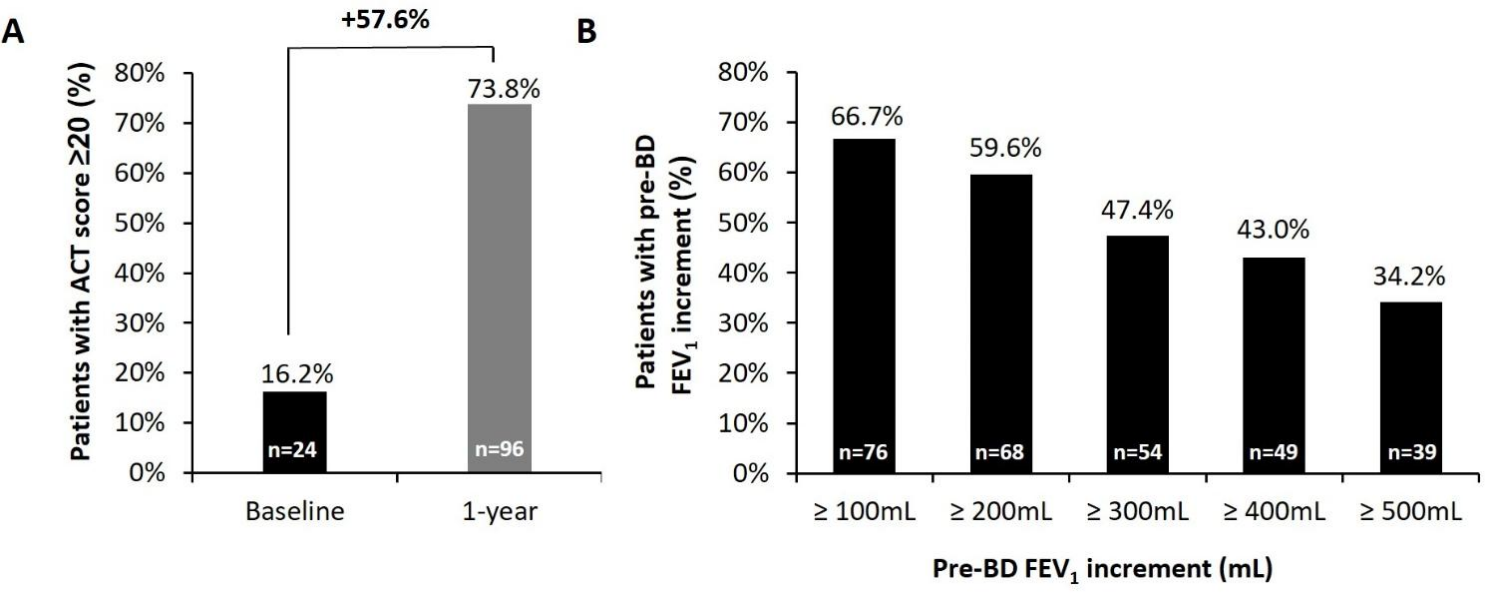
Improvements in asthma control and lung function in the OP. **(A)** Change in the proportion of patients with an ACT score ≥ 20 from baseline to 1 year of follow-up. **(B)** Proportion of patients who achieved increases in pre-BD FEV_1_ over a certain threshold after 1 year of follow-up. ACT, asthma control test; BD, bronchodilator; FEV_1_, forced expiratory volume in 1 s

Asthma symptom control improved over the year of FUP as confirmed by a mean (SD) increase in the ACT score of 6.6 (6.0) points (Table 3, Additional file 1: Figure S2A). The proportion of patients achieving an ACT score ≥ 20 points was 73.8% at 1-year FUP (Fig. 2A), and 72.1% of the OP had an increase in the ACT score of ≥ 3 points (Table 3).

In terms of lung function, the mean (SD) increase in pre-BD FEV_1_ was 331 (413) mL at 1-year FUP, with 66.7% and 34.2% of patients achieving an increase in the pre-BD FEV_1_ of ≥ 100 mL or ≥ 500 mL, respectively (Table 3; Fig. 2B, Additional file 1: Figure S2B). A pre-BD FEV_1_ ≥ 80% of predicted was observed in 46.3% of patients (Table 3, Additional file 1: Figure S3A). However, among patients with baseline FEV_1_ < 80% or < 60%, this proportion was reduced to 34.1% (n = 30) and 13.6% (n = 6), respectively.

**Fig. 3:**
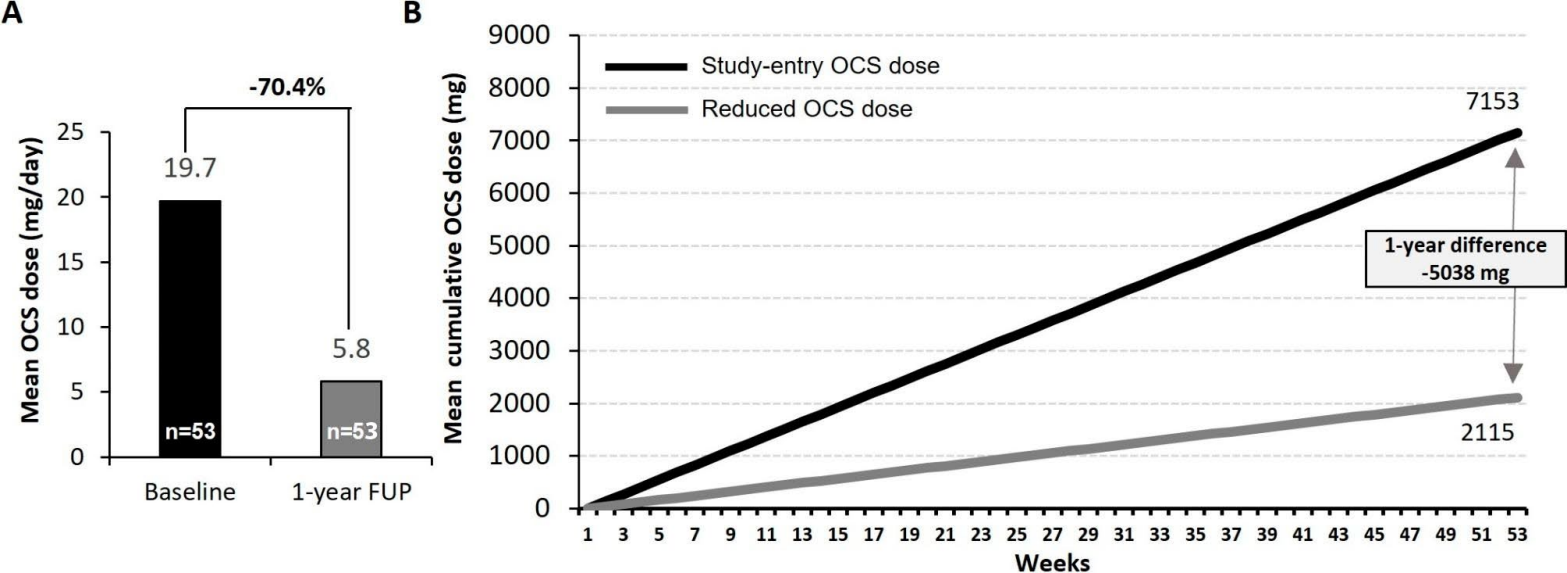
Use of OCS over time in OCS-dependent patients (n = 53). **(A)** Mean OCS dose reduction at 1 year of FUP. **(B)** Estimated mean cumulative OCS exposure over 1 year of FUP for patients continuing on study-entry mean OCS dose compared with patients treated with benralizumab and reducing the OCS dose after 1 year of FUP. FUP, follow-up; OCS, oral corticosteroids

Finally, over 1 year of FUP, a total of 28 out of 53 OCS-dependent patients (52.8%) on benralizumab treatment were able to completely withdraw OCS. The median [Q1-Q3] daily dose of maintenance OCS fell from 15 [6.9–29.0] mg at baseline to 0.0 [0.0-8.3] mg and mean daily OCS dose reduction was 70.5% at 1-year FUP, with 62.3% of patients achieving a daily dose reduction of ≥ 50% (Table 4; Fig. 3A). Mean cumulative OCS exposure during 1 year was estimated at 2115 mg for patients receiving benralizumab and at 7153 mg if patients had remained on the baseline OCS dosage, which translated into an estimated mean reduction in cumulative OCS exposure of 5038 mg over 1 year (Fig. 3B).

**Table 4:** Oral corticosteroid (OCS) reduction in OCS-dependent patients receiving benralizumab treatment at 1 year of follow-up

	Baseline	1-year FUP
Corticosteroid (CS)-dependent patients, n/N (%)	53/182 (29.1%)	25/200 (12.5%)
*Daily OCS dose (mg), mean (SD)*	19.7 (15.8)	5.8 (9.4)
*Daily OCS dose (mg), median [Q1-Q3]*	15 [6.3–29.0]	0.0 [0.0-8.3]
**Change in OCS use**		
Patients achieving an OCS dose reduction ≥ 50%, n/N (%)		33/53 (62.3%)
Patients achieving complete OCS withdrawal, n/N (%)		28/53 (52.8%)
Percentage reduction in mean daily OCS dose		70.4%
Percentage reduction in median daily OCS dose		100.0%

Corticosteroid-dependent patients were defined as those who received maintenance systemic corticosteroid treatment for at least 3 months, within the 12 months prior to the index date

CS, corticosteroids; FUP, follow-up; quartiles 1 and 3 (Q1-Q3); OCS, oral corticosteroids; SD, standard deviation

**Fig. 4:**
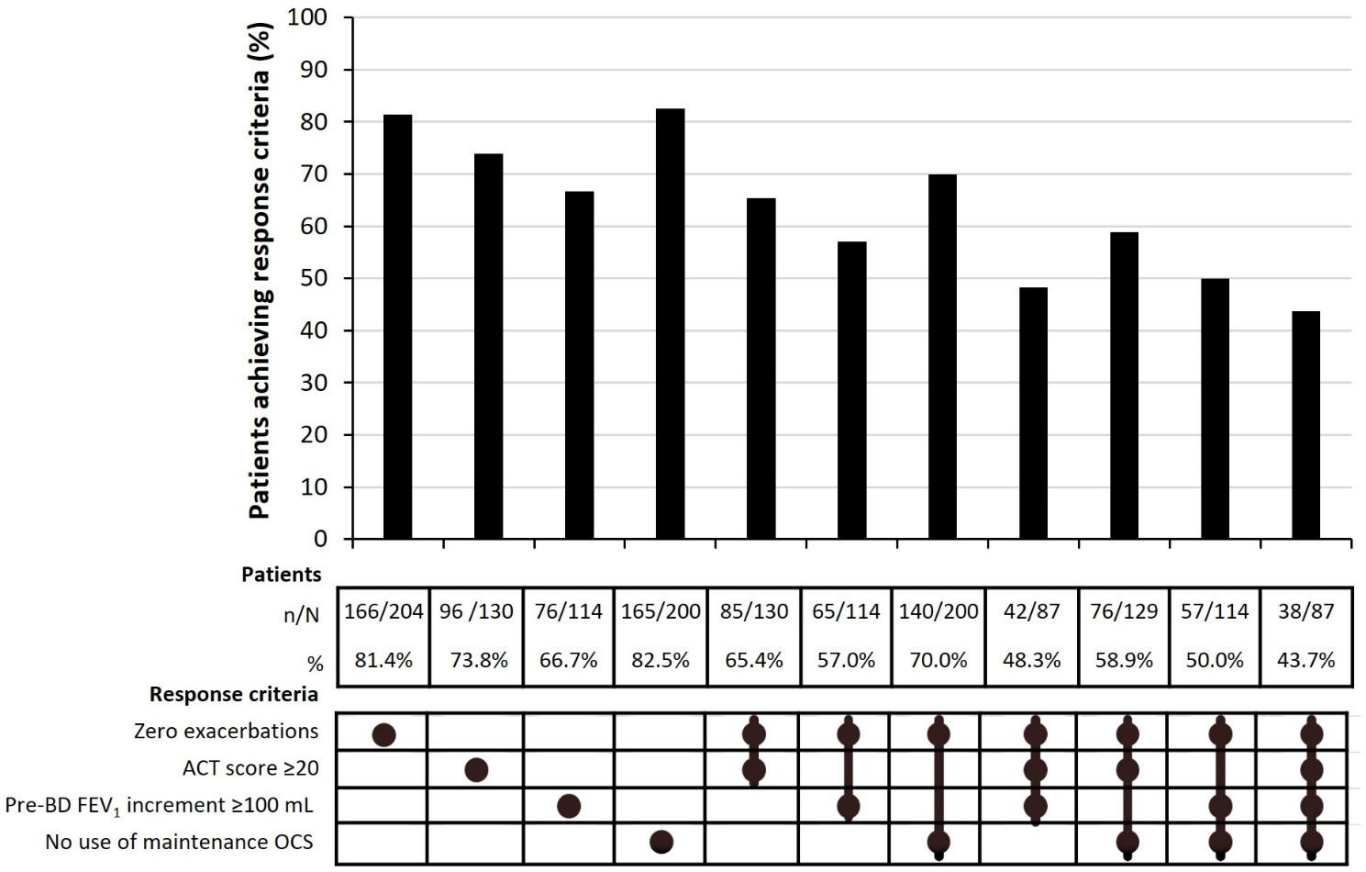
Response criteria fulfilled after 1-year. UpsSet plot showing the number and proportion of patients who met 1–4 key criteria for clinical response to benralizumab. Each column represents the percentage of patients who met one or several pre-defined criteria after 1 year of follow-up. Percentages are calculated over the total number of patients with valid data. Key clinical outcomes included absence of severe exacerbations, asthma symptom control as defined by an ACT score ≥ 20, lung function improvement as defined by an increase in FEV_1_ ≥ 100 mL and no use of OCS. “n” represents the number of patients who met the specified criteria, while “N” represents the total number of patients with available data. ACT, asthma control test; BD, bronchodilator; FEV_1_, forced expiratory volume in 1 s; OCS, oral corticosteroids

**Fig. 5:**
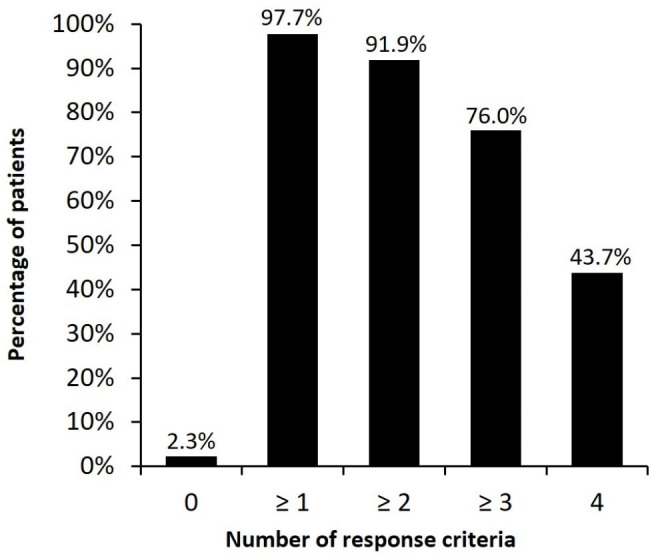
Proportion of patients who fulfilled response criteria after 1-year of follow-up. Percentage of patients on benralizumab treatment represented in categories according to the number of different response criteria fulfilled after 1-year of follow up

Finally, we assessed the proportion of patients reaching any level of response to benralizumab and clinical remission over the 1-year FUP, based on the achievement of at least one of the following key response components: no exacerbations; total maintenance OCS withdrawal; pre-BD FEV_1_ increase ≥ 100 mL from baseline; and ACT score ≥ 20. In terms of response and based on the published definition of super-responders (patients achieving zero exacerbations and total OCS withdrawal), we found that 70% of patients corresponded to this category. More importantly, 43.7% of our cohort of SEA patients would have achieved clinical remission, as they fulfilled the four pre-specified criteria (Fig. 4). When taking into consideration the total number of response criteria analysed for the study population that patients on benralizumab treatment could potentially fulfil, most of the study population fulfilled at least one, two or three of the response criteria (98%, 92% and 76%, respectively) (Fig. 5). Complete data on response criteria and the proportion of patients who achieved response are presented in Additional file 1: Figure S4.

### Baseline characteristics and response to benralizumab in SEA patients with and without concomitant CRSwNP

Demographic characteristics and clinical data of patients with and without concomitant CRSwNP were analysed at baseline and at 1-year FUP. The subgroup with CRSwNP presented a numerically higher proportion of men and lower proportion of patients with the allergic asthma phenotype or who were current or former smokers (Additional file 1: Table S2). Numerically higher median [Q1-Q3] FeNO levels (45.5 [19.0–81.0] ppb) and eosinophil counts (600 [320–870] cells/µL) were also confirmed in this subgroup (versus 34 [19.8–53] ppb and 410 [200–700] cells/µL, respectively, for the subgroup without CRSwNP), and were also greater than those observed in the OP (Additional file 1: Table S3).

After 1-year of FUP, no remarkable differences were observed between the subgroups with and without CRSwNP in terms of a decrease in T2 inflammation biomarkers (Additional file 1: Table S3), which did not differ from the overall rates of reduction observed in the OP.

With respect to clinical outcomes, the overall reduction in severe exacerbations observed at 1-year FUP from baseline was similar, independently of the presence of concomitant CRSwNP in patients treated with benralizumab (approximately 86% in both subgroups), in line with the rate of reduction in the OP (Additional file 1: Table S4, Figure S1B and 1 C). However, a slightly higher percentage of patients with CRSwNP experienced a ≥ 50% reduction in exacerbations (93.8% versus 90.8% of patients without concomitant CRSwNP), numerically higher than even that in the OP. The use of healthcare resources was higher among patients with concomitant CRSwNP, who experienced a 96.7% reduction in the rate of hospitalizations and 88% reduction in visits to the ED (versus 88.6% and 85%, respectively, in patients without concomitant CRSwNP) (Additional file 1: Table S4).

Regarding asthma symptom control, a similar mean increase of 6.5–6.7 points in the ACT score was observed in patients independently of the presence of concomitant CRSwNP (Additional file 1: Table S4, Figure S2A), and an ACT score ≥ 20 was observed in approximately 74% of patients in both subgroups (Additional file 1: Table S4, Figure S5). However, it should be noted that a numerically higher percentage of patients with CRSwNP achieved an increase in the ACT score of ≥ 3 points (76.9%) while 70% of patients without CRSwNP and 69.2% of the OP achieved this increase (Additional file 1: Table S4, Figure S6).

In terms of lung function, improvements were confirmed in both patient subgroups, although higher increases were observed in patients with CRSwNP. The mean (SD) increase in pre-BD FEV_1_ was 426 (420) mL in the presence of CRSwNP and 277 (401) mL in the subgroup without concomitant CRSwNP (Additional file 1: Table S4, Figure S2B). This CRSwNP subgroup had a greater proportion of patients with a pre-BD FEV_1_ ≥ 80% at 1-year FUP (52.2% versus 43.2% of patients without concomitant CRSwNP) (Additional file 1: Table S4, Figure S3B and C).

After 1-year of FUP of patients receiving benralizumab, the proportion of maintenance OCS-free patients increased in both groups. The proportion of OCS-dependent patients was lower in the subgroup with CRSwNP (6.9%), while 14.8% of patients without concomitant CRSwNP were still dependent on maintenance OCS. A numerically greater percentage of patients with CRSwNP completely withdrew maintenance OCS (70.6%), while 47.2% of patients without concomitant CRSwNP were also able to discontinue this therapy. The number of patients who achieved this outcome was higher with respect to the percentage for the OP (52.8%). Patients with concomitant CRSwNP reduced the median [Q1-Q3] daily dose of maintenance OCS from 10 [5.0–32.0] mg at baseline to 0.0 [0.0–5.0] and had a 87.5% mean reduction in the daily OCS dose after 1-year FUP, with 58.3% of patients achieving a daily dose reduction of ≥ 50% (Additional file 1: Table S5, Figure S7A). Mean cumulative OCS exposure in 1 year was estimated at 965 mg for patients with concomitant CRSwNP receiving benralizumab and 7870 mg if patients had remained on the baseline OCS dosage, which translated into an estimated mean reduction in cumulative OCS exposure of 6905 mg over 1 year (Additional file 1: Figure S7B).

In summary, after 1 year of FUP, similar outcomes were observed in terms of the reduction in exacerbations and increase in the ACT score, regardless of the presence of concomitant CRSwNP, and a higher proportion of patients with CRSwNP achieved a ≥ 50% reduction in exacerbations and an increase of ≥ 3 points in the ACT score. A greater reduction in the number of hospitalizations and visits to the ED, overall improvements in pre-BD FEV_1_ and a reduction in the number of OCS-dependent patients and daily OCS dose were observed in patients with CRSwNP.

## Discussion

The ORBE II study, as part of the XALOC programme, is one of the largest real-world studies on benralizumab at international level and the largest one presented in Spain to date. The first real-life studies assessing the therapeutic effect of benralizumab in small SEA patient cohorts confirmed a rapid gain in asthma control and lung function, a significant reduction or elimination of exacerbations and maintenance OCS use, and a decrease in the number of ED visits [30–34]. More importantly, data from these studies confirmed that the improvement in all these clinical outcomes was sustained and even increased over time. Several findings from real-world studies within the XALOC programme supported the effectiveness of benralizumab and its capacity to induce clinical remission in patients [35–38].

The profile of our ORBE II cohort was representative of the SEA population. This was mainly defined by: high levels of T2 inflammation biomarkers (73% of patients presented eosinophil counts ≥ 300 cells/µL and 37% of patients had FeNO levels ≥ 50ppb); CRSwNP as the most common comorbidity (36.8% of patients); late asthma onset (mean age of asthma onset was ≥ 30 years), frequent exacerbations (≥ 2 in a year); compromised lung function; poor symptom control; and high OCS use. Most of these features were indicative of a strong eosinophilic phenotype [2, 8, 39] and, moreover, were valuable predictors of enhanced responses to benralizumab, as confirmed by previous real-world evidence [25, 27, 30, 40]. High eosinophil counts and FeNO concentrations are indicators of increased exacerbation risk [41]. After 1 year of FUP, we observed a significant drop in both biomarkers in patients on benralizumab. These findings are consistent with prior evidence [36], and could be closely related to the mechanisms underlying the antibody-dependent cellular cytotoxicity of benralizumab on eosinophils and basophils [12, 42].

Interestingly, a large subset of patients in the ORBE II cohort (1 out of 4) presented low eosinophil counts (< 300 cells/µL) at baseline, a particular subset of patients in whom a positive response to benralizumab was previously shown [25, 32]. It is important to note that the use of high-dose ICS or OCS has been related to reduced eosinophil levels [43, 44], and that these levels may vary over time [45], so lower eosinophil counts at one time point may not be enough to characterise eosinophilic patients.

According to data collected on previous treatment patterns, most of the OP of our study (almost 70%) was biologic-naïve and showed marked eosinophilia, more frequent exacerbations and worse asthma control than switch patients (data not shown). Switch patients had discontinued other previous biological treatments mostly due to lack of response. The overall improved outcomes observed after 1 year of FUP were in line with previous findings showing that benralizumab could even benefit SA patients unresponsive to other biologics targeting the IL-5 or IgE pathways [36, 46–49]. However, naïve patients showed a higher response, thus highlighting the importance of correct patient phenotyping and choice of biologic.

The objectives of SEA treatment have been clearly defined by clinical guidelines, and recommend a stepwise therapeutic strategy aimed at reducing the risk of severe attacks and optimizing symptom control, as well as monitoring the response to therapy to confirm that clinical objectives are achieved [1, 3]. Asthma exacerbations are probably the main contributors to the burden of SA and are commonly defined as the requirement for systemic maintenance treatment with OCS and their association with hospitalization or ED visits and lung function deterioration [50]. Thus, the reduction or even elimination of exacerbations, which should also have a positive impact in terms of reducing OCS use, is one of the key goals of SA management [19, 51]. In our cohort, 81.4% of patients on benralizumab achieved zero exacerbations after 1 year of FUP and we found a remarkable 85.6% reduction in severe exacerbations, which is consistent with the results of published analyses of the XALOC programme [38, 52].

This reduction in severe exacerbations was achieved in a context in which the median [Q1-Q3] daily dose of maintenance OCS in cortico-dependent patients was drastically reduced from 15 [6.3–29.0] to 0.0 [0.0-8.3] mg, in line with reports from other cohorts of SEA patients treated with benralizumab [30, 36, 53]. For decades, OCS have been a valuable long-term treatment in the management of uncontrolled SA patients at risk of repeated severe exacerbations or with poor asthma control. Because of the known OCS-related adverse effects and their associated health-related costs in the short and long term [54–57], current clinical guidelines recommend biologics as the preferred and safer alternative to long-term OCS [3], and suggest that a reduction or even total elimination of OCS be pursued as a fundamental therapeutic goal in SA [7, 58, 59].

The OCS-sparing effect of benralizumab in SEA patients was confirmed in the ZONDA clinical trial, and even expanded in the PONENTE study, which showed that this benefit was independent of the blood eosinophil count at baseline [60, 61]. These data were further supported by real-world evidence [15, 16, 30, 62]. In the ORBE II cohort, 53% of OCS-dependent patients on benralizumab discontinued OCS after 1 year of FUP. Furthermore, reduction or elimination of exacerbations help to minimize OCS use, as each exacerbation treated with OCS contributes to its detrimental accumulation.

A real-world study in Spain estimated the 1-year cumulative OCS dose in 2019 to be more than 1 g per SA patient [63], showing that there is still high OCS overuse even with the current availability of alternative therapeutic approaches such as biologics. In this sense, a recent Spanish multidisciplinary consensus highlights the need to monitor the cumulative OCS dose, and strongly recommends that patients receiving a cumulative OCS dose of ≥ 0.5 g per year should be provided with alternative therapeutic options [7]. Our own analyses estimated that the cumulative OCS dose over 1 year of FUP, if patients had remained on their baseline dose, would have been 7.1 g, which is seven times higher than the recommended annual limit of 0.5 to 1 g per year.

Benralizumab-mediated OCS tapering has been shown to effectively reduce the cumulative OCS dosage over time [18]. Our estimations pointed to a mean reduction in cumulative OCS exposure of 5.0 g after 1-year FUP in our cohort of patients on benralizumab. It is important to highlight that these estimations did not take into account the reduction in OCS use associated with the drop in exacerbations while on benralizumab treatment.

As already mentioned, improved lung function and asthma symptom control are other fundamental goals of SA treatment. The mean (SD) increase in pre-BD FEV_1_ of 331 (413) mL confirmed in our cohort after 1 year of FUP was even better than the results reported in the pivotal SIROCCO [13] and CALIMA [14] trials, which confirmed an FEV_1_ gain of 159 mL and 116 mL vs. placebo, respectively, after benralizumab. This was accompanied by a remarkable increase in the ACT score of a mean of almost 7 points. Moreover, approximately three out of four patients in the ORBE II cohort achieved an ACT score ≥ 20, which is an indicator of well-controlled asthma [64].

The assessment of response of SA patients to biologics must be multidimensional [65]. Most published definitions of response and asthma clinical remission rely, in fact, on composite measures of treatment response. Up to four measurable domains are the most frequently considered at the time to evaluate the response of SA to treatment (severe exacerbations, asthma control, OCS use and lung function), although the stringency of the number of criteria and cut-offs established to determine the degree of response or clinical remission is more controversial [4, 10, 11, 66]. The real-world studies conducted by Kavanagh et al. [30] and Jackson et al. [36] identified 39% and 57.2% of super-responders to benralizumab in their respective cohorts according to the combination of two pre-defined outcomes: zero exacerbations and no maintenance OCS use. Taking into account the same definition, integrated analyses of the international real-world XALOC-1 study estimated the proportion of super-responders to be 60% [38]. Miralles-López et al. [67] reported that 63% of patients met these super-responder criteria. Additionally, the real-world study by Padilla-Galo et al. [68] found that 59.1% of patients met this super-responder definition. Based on these standards, a higher proportion of patients (7 out of 10) in our ORBE II cohort were identified as super-responders to benralizumab after 1-year FUP.

A more stringent definition was proposed by the Spanish Severe Asthma Consensus [4]. The complete response criteria included all the following: absence of exacerbations; no maintenance OCS use; ACT score ≥ 20 points; and pre-BD FEV_1_ ≥ 80% of predicted. Following this definition, Miralles-López et al. [67] and Padilla-Galo et al. [68] identified 36.6% and a 27.3% of complete responders in their cohorts, respectively.

These criteria were in line with the general framework for asthma clinical remission proposed by Menzies-Gow et al. (2020) [10], which required the stabilisation of lung function and the absence for at least 12 months of significant asthma symptoms and severe exacerbations. The key variables of asthma remission included the elimination of asthma exacerbations, complete withdrawal of OCS, and improvement in asthma control and lung function. Based on this definition, we found that almost half of our cohort of SEA patients receiving benralizumab treatment (43.7%) fulfilled four established criteria defining clinical remission. It should be noted that most of our OP treated with benralizumab met at least 1–3 of the response criteria after 1-year FUP. Taking into account all evaluated criteria, an FEV1 ≥ 80% is the criterion that is more difficult to achieve. In fact, the results in the subset of patients with baseline FEV_1_ below 80% and 60% suggests that difficulty in achieving such criterion increases with a more compromised lung function. Therefore, this may be due to the deterioration in lung function that occurred before treatment initiation and could indicate the need to start biologic treatment earlier in the course of the disease.

Data from clinical trials have suggested that SA patients presenting concomitant CRSwNP achieved enhanced responses to benralizumab and improvements in parameters linked to this comorbidity, such as SNOT-22 [25, 27]. This was also confirmed in the real-world setting [35, 53, 69]. In our cohort, we observed that clinically relevant responses to benralizumab were confirmed regardless of the presence of concomitant CRSwNP. Nevertheless, improved clinical outcomes in some parameters such as lung function and reduction in OCS use were observed in the subset of patients with concomitant CRSwNP, supporting the particular benefits of benralizumab in this SEA patient subpopulation.

Our study has limitations that are inherent to its retrospective and real-world nature. First of all, a single-arm study does not allow to demonstrate the benefit of a specific treatment versus a standard of care approach. Incomplete information or loss of data during patient follow-up did not allow us to provide a complete characterization of clinical outcomes in the subgroup analyses. Additionally, no sensitivity analyses were performed to discard any bias in results due to heterogeneity in selection criteria or procedures and data collection among sites. Despite these limitations, the strengths of this study include the large sample size and high number of specialised asthma units throughout Spain and the lack of highly restrictive inclusion criteria which make these results representative of the SEA real-world population treated with benralizumab. As an additional strength, this work includes information on innovative aspects such as cumulative OCS dose and asthma remission, for which there are few published studies at present.

## Conclusions

In summary, this extensive real-world ORBE II study described the clinical and demographic characteristics of adult SEA patients receiving benralizumab in routine clinical practice conditions in Spain. The features of this cohort of patients were representative of SA patients with an eosinophilic phenotype, mainly characterised by a high presence of T2 inflammation biomarkers, late onset of asthma, comorbidities including CRSwNP, high rate of exacerbations, use of maintenance OCS, and poor asthma control. In this population, a high proportion of patients treated with benralizumab were defined as super-responders and moreover, met all key criteria for asthma clinical remission. The clinically meaningful benefits of benralizumab confirmed in the overall study cohort were also found in the subgroup of patients with concomitant CRSwNP, who achieved similar or even enhanced responses to treatment. These results highlight the clinical benefits of benralizumab in a real-world setting and show that it allows clinical objectives and even clinical remission to be accomplished.

### Electronic supplementary material

Below is the link to the electronic supplementary material.

Supplementary Material 1

## Data Availability

The datasets used and analysed during the current study may be obtained in accordance with AstraZeneca’s data sharing policy, described at https://astrazenecagrouptrials.pharmacm.com/ST/Submission/Disclosure.

## Abbreviations

ACT: Asthma control test
CRSwNP: Chronic rhinosinusitis with nasal polyposis
FeNO: Fraction of exhaled nitric oxide
FEV_1_: Forced expiratory volume in 1 s
FUP: Follow-up
OCS: Oral corticosteroids
OP: Overall population
Q1: Quartile 1 (25%)
Q3: Quartile 3 (75%)
SA: Severe asthma
SEA: Severe eosinophilic asthma
SD: Standard deviation
