# Supplementary material for: Achieving clinical outcomes with benralizumab in severe eosinophilic asthma patients in a real-world setting: ORBE II study
Source: Respir Res. 2023 Sep 28;24:235. doi: 10.1186/s12931-023-02539-7 (PMC10540395; doi:10.1186/s12931-023-02539-7)

Additional File 1

**ACHIEVING CLINICAL OBJECTIVES WITH BENRALIZUMAB IN SEVERE EOSINOPHILIC ASTHMA PATIENTS IN A REAL-WORLD SETTING: ORBE II STUDY**

**Table of contents:**

**Table S1** - Patients discontinuing benralizumab within 1-year of follow-up and reasons for discontinuation (page 3).

**Table S2** - Baseline demographic characteristics of SEA patients with or without concomitant CRSwNP (pages 5-6).

**Table S3** - Biomarkers dynamics in the overall population and in the subgroups of SEA patients with or without concomitant CRSwNP receiving benralizumab treatment at 1 year of follow-up (page 4).

**Table S4** - Baseline clinical characteristics and clinical outcomes of SEA patients with or without concomitant CRSwNP receiving benralizumab treatment at 1 year of follow-up (pages 7-8).

**Table S5** - Oral corticosteroids (OCS) reduction in OCS-dependent patients with or without concomitant CRSwNP receiving benralizumab treatment at 1-year of follow-up (page 9).

**Figure S1** - Change in the mean number of severe exacerbations from baseline to 1 year of follow-up (page 10).

**Figure S2** - Improvements in asthma control and lung function n in the OP and in the subgroups of patients with or without comorbid CRSwNP (page 11).

**Figure S3** - Proportion of patients with pre-BD FEV1 ≥80% of predicted at baseline and at 1 year of follow-up (page 12).

**Figure S4** - UpSet plot showing the number and proportion of patients who met one or several of the pre-defined response criteria to benralizumab (page 13).

**Figure S5** - Proportion of patients with an ACT score ≥20 points at baseline and at 1 year of follow-up according to the presence or not of comorbid CRSwNP (page 14).

**Figure S6** - Proportion of patients with an increase in the ACT score ≥3 points at 1 year of follow-up (page 15).

**Figure S7** - Change in use of maintenance oral corticosteroids (OCS) over time in OCS-dependent patients with comorbid CRSwNP (n = 17) (page 16).

**Table S1.** Patients discontinuing benralizumab within 1-year of follow-up and reasons for discontinuation.

|  | **Overall Population**  **N = 204** | **Patients with comorbid CRSwNP**  **N = 75** | **Patients with no comorbid CRSwNP**  **N = 129** |
| --- | --- | --- | --- |
| **Discontinuation due to any reason, n/N (%)** | 11/204 (5.4%) | 3/75 (4.0%) | 8/129 (6.2%) |
| Reasons for discontinuation, n/N (%) |  |  |  |
| *Lack of response* | 4/204 (2.0%) | 2/75 (2.7%) | 2/129 (1.6%) |
| *Patient’s decision* | 3/204 (1.5%) | - | 3/129 (2.3%) |
| *Other* | 4/204 (2.0%) | 1/75 (1.3%) | 3/129 (2.3%) |

Due to the nature of this real-world study, sample sizes vary due to missing, unavailable and/or not valid data. “N” represents the total number of patients with valid data. All values were calculated over the total of patients with valid data.

CRSwNP, chronic rhinosinusitis with nasal polyposis.

**Table S2.** Baseline demographic characteristics of SEA patients with or without concomitant CRSwNP.

| **Parameters at baseline** | **Patients with comorbid CRSwNP**  **N = 75** | **Patients with no comorbid CRSwNP**  **N = 129** |
| --- | --- | --- |
| **Age (years), mean (SD)** | 54.0 (12.2) | 57.7 (12.4) |
| **Women, n/N (%)** | 38/75 (50.7%) | 89/129 (69.0%) |
| **BMI (Kg/m^2^), mean (SD)^a^** | 26.3 (4.9) | 29.2 (6.8) |
| *Obese (BMI ≥30 Kg/m2), n/N (%)* | 14/71 (19.7%) | 40/117 (34.2%) |
| **Age at asthma onset (years), mean (SD)** | 34.2 (14.9) | 34.6 (17.3) |
| **Asthma duration (years), mean (SD)** | 15.3 (11.9) | 14.9 (13.3) |
| **Allergic asthma, n/N (%)^b^** | 19/75 (25.3%) | 49/129 (38.0%) |
| **Smoking history, n/N (%)^c^** |  |  |
| *Non-smoker* | 50/75 (66.7%) | 78/128 (60.9%) |
| *Former smoker* | 24/75 (32.0%) | 45/128 (35.2%) |
| *Smoker* | 1/75 (1.3%) | 5/128 (3.9%) |
| **Comorbidities, n/N (%)** |  |  |
| *CRS with NP* | 75/75 (100%) | 0/129 (0.0%) |
| *Obesity* | 18/75 (24.0%) | 44/129 (34.1%) |
| *Gastroesophageal reflux* | 15/75 (20.0%) | 27/129 (20.9%) |
| *Dyslipidemia* | 10/75 (13.3%) | 26/129 (20.2%) |
| *CRS without NP* | 0/75 (0.0%) | 35/129 (27.1%) |
| *Osteoporosis* | 11/75 (14.7%) | 21/129 (16.3%) |
| *Arterial hypertension* | 8/75 (10.7%) | 24/129 (18.6%) |
| *Sleep apnea syndrome* | 6/75 (8.0%) | 20/129 (15.5%) |
| *Type II diabetes mellitus* | 3/75 (4.0%) | 15/129 (11.6%) |
| *Depression* | 7/75 (9.3%) | 10/129 (7.8%) |
| *COPD* | 1/75 (1.3%) | 13/129 (10.1%) |
| *Anxiety* | 1/75 (1.3%) | 12/129 (9.3%) |
| *Hypothyroidism* | 7/75 (9.3%) | 5/129 (3.9%) |

Due to the nature of this real-world study, sample sizes vary due to missing, unavailable and/or not valid data. “N” represents the total number of patients with valid data. All values were calculated over the total of patients with valid data. ^a^Data missing from 4 patients with CRSwNP and from 12 patients with no CRSwNP. ^b^As determined by the investigator. ^c^Data missing from 1 patient with no CRSwNP.

BMI, body mass index; COPD, chronic obstructive pulmonary disease; CRSwNP, chronic rhinosinusitis with nasal polyposis; NP, nasal polyposis; SD, standard deviation.

**Table S3.** Biomarkers dynamics in the overall population and in the subgroups of SEA patients with or without concomitant CRSwNP receiving benralizumab treatment at 1 year of follow-up.

| **Parameters** | **Overall Population** | | **Patients with comorbid CRSwNP** | | **Patients with no comorbid CRSwNP** | |
| --- | --- | --- | --- | --- | --- | --- |
|  | **Baseline** | **1-year FUP** | **Baseline** | **1-year FUP** | **Baseline** | **1-year FUP** |
| **Blood eosinophil counts** | **N = 197** | **N = 138** | **N = 74** | **N = 52** | **N = 123** | **N = 86** |
| Eosinophil counts (cells/ µl),  median [Q1-Q3] | 500 [220-750] | 0 (0-0) | 600 [320-870] | 0 [0-0] | 410 [200-700] | 0 [0-0] |
| *≥300 cells/µL, n/N (%)* | 143/197 (72.6%) | 2/138 (1.5%) | 58/74 (78.4%) | 2/52 (3.9%) | 85/123 (69.1%) | 0/86 (0.0%) |
| *≥500 cells/µL, n/N (%)* | 100/197 (50.8%) | 1/138 (0.7%) | 45/74 (60.8%) | 1/52 (1.9%) | 55/123 (44.7%) | 0/86 (0.0%) |
| **Total serum IgE levels** | **N = 144** | **N = 57** | **N = 60** | **N = 24** | **N = 84** | **N = 33** |
| IgE levels (IU/mL), median [Q1-Q3] | 163.5 [49.8-432] | 155 [81.7-415] | 148 [48.5-446] | 206.5 [96.2-870.5] | 192 [54.9-418.5] | 143 [65-247] |
| **FeNO** | **N = 120** | **N = 88** | **N = 54** | **N = 31** | **N = 66** | **N = 57** |
| FeNO (ppb), median [Q1-Q3] | 36.8 [19.2-64] | 24.9 [15.9-55.3] | 45.5 [19.0-81.0] | 32 [19.5-54] | 34 [19.8-53] | 23 [15-54] |
| *FeNO < 25 ppb, n/N (%)* | 44/120 (36.7%) | 44/88 (50.0%) | 18/54 (33.3%) | 12/31 (38.7%) | 26/66 (39.4%) | 32/57 (56.1%) |
| *FeNO ≥ 25 ppb < 50 ppb, n/N (%)* | 30/120 (25.0%) | 18/88 (20.5%) | 10/54 (18.5%) | 11/31 (35.5%) | 20/66 (30.3%) | 7/57 (12.3%) |
| *FeNO ≥ 50 ppb, n/N (%)* | 46/120 (38.3%) | 26/88 (29.6%) | 26/54 (48.1%) | 8/31 (25.8%) | 20/66 (30.3%) | 18/57 (31.6%) |

Due to the nature of this real-world study, sample sizes vary due to missing, unavailable and/or not valid data. “N” represents the total number of patients with valid data. All values were calculated over the total of patients with valid data.

CRSwNP, chronic rhinosinusitis with nasal polyposis; FeNO, fractional exhaled nitric oxide; FUP, follow up; IgE, immunoglobulin E; quartiles 1 and 3 [Q1-Q3]; IU, international units; ppb, parts per billion; SD, standard deviation.

**Table S4.** Baseline clinical characteristics and clinical outcomes of SEA patients with or without concomitant CRSwNP receiving benralizumab treatment at 1 year of follow-up.

| **Parameters** | **Patients with comorbid CRSwNP**  **Total patients = 75** | | **Patients with no comorbid CRSwNP**  **Total patients = 129** | |
| --- | --- | --- | --- | --- |
|  | **Baseline** | **1-year FUP** | **Baseline** | **1-year FUP** |
| **Severe exacerbations** |  |  |  |  |
| Severe exacerbations, mean (SD) | 2.3 (1.7) | 0.33 (0.81 | 2.7 (2.6) | 0.37 (1.07) |
| Patients with zero severe exacerbations, n/N (%) | 11/75 (14.7%) | 61/75 (81.3%) | 20/ 129 (15.5%) | 105/129 (81.4%) |
| **Changes in severe exacerbations** |  |  |  |  |
| Patients with severe exacerbations reduction, n/N (%) | - | 60/64 (93.8%) | - | 101/109 (92.7%) |
| Patients achieving ≥ 50% reduction in severe exacerbations, n/N (%) | - | 60/64 (93.8%) | - | 99/109 (90.8%) |
| Percentage reduction in severe exacerbations | - | 86.7% | - | 86.3% |
| **Asthma-related use of healthcare resources** |  |  |  |  |
| Patients with no hospitalizations, n/N (%) | 62/74 (83.8%)^a^ | 74/75 (98.7%) | 97/129 (75.2%) | 122/129 (94.6%) |
| Hospitalizations, mean (SD) | 0.3 (0.6)^b^ | 0.01 (0.12) | 0.5 (1.1)^c^ | 0.06 (0.26) |
| Patients with no ED visits, n/N (%) | 44/75 (58.7%) | 71/75 (94.7%) | 74/129 (57.4%) | 117/129 (90.7%) |
| ED visits, mean (SD) | 0.7 (1.2) | 0.08 (0.36) | 1.0 (2.1) | 0.15 (0.60) |
| **Changes in use of asthma-related healthcare resources** |  |  |  |  |
| Percentage reduction in average hospitalizations | - | 96.7% | - | 88% |
| Percentage reduction in ED visits | - | 88.6% | - | 85% |
| **Lung function** |  |  |  |  |
| Pre-BD FEV_1_ (mL), mean (SD) | 2124 (839)^d^ | 2513 (808)^e^ | 1783 (735)^f^ | 2007 (756)^g^ |
| Pre-BD FEV_1_ (% predicted), mean (SD) | 68.4 (19.5)^h^ | 80.3 (18.1)^i^ | 66.6 (21.9)^j^ | 77.9 (24.2)^k^ |
| Patients with pre-BD FEV_1_ ≥ 80%, n/N (%) | 20/62 (32.3%) | 24/46 (52.2%) | 30/108 (27.8%) | 38/88 (43.2%) |
| **Changes in lung function** |  |  |  |  |
| Increase in pre-BD FEV_1_ (mL), mean (SD) | - | 426 (420) | - | 277 (401) |
| Pre-BD FEV_1_ increment ≥ 100mL, n/N (%) | - | 30/41 (73.2%) | - | 46/73 (63.0%) |
| Pre-BD FEV_1_ increment ≥ 200mL, n/N (%) | - | 30/41 (73.2%) | **-** | 38/73 (52.1%) |
| Pre-BD FEV_1_ increment ≥ 300mL, n/N (%) | - | 23/41 (56.1%) | - | 31/73 (42.5%) |
| Pre-BD FEV_1_ increment ≥ 400mL, n/N (%) | **-** | 22/41 (53.7%) | - | 27/73 (37.0%) |
| Pre-BD FEV_1_ increment ≥ 500mL, n/N (%) | **-** | 19/41 (46.3%) | **-** | 20/73 (27.4%) |
| **Asthma control** |  |  |  |  |
| ACT score, mean (SD) | 14.8 (5.2)^i^ | 21.2 (4.9)^k^ | 13.7 (5.1)^l^ | 20.8 (4.7^)m^ |
| Patients with an ACT score ≥20, n/N (%) | 10/54 (18.5%)^i^ | 37/50 (74.0%)^k^ | 14/94 (14.9%)^l^ | 59/80 (73.8%)^j^ |
| Patients with an ACT score increment ≥3, n/N (%) | - | 30/39 (76.9%) | - | 45/65 (69.2%) |
| Increase in ACT score, mean (SD) |  | 6.7 (6.2) |  | 6.5 (5.9) |

Due to the nature of this real-world study, sample sizes vary due to missing, unavailable and/or not valid data. “N” represents the total number of patients with valid data. All values were calculated over the total of patients with valid data.

Data missing/not valid data from: ^a^1 patient, ^b^12 patients; ^c^20 patients; ^d^18 patients; ^e^30 patients; ^f^47 patients; ^g^13 patients; ^h^29 patients; ^i^21 patients; ^j^41 patients; ^k^25 patients; ^l^35 patients; ^m^49 patients.

ACT, asthma control test; CRSwNP, chronic rhinosinusitis with nasal polyposis; ED, emergency department; FEV_1_, forced expiratory volume in the first second; FUP, follow up; pre-BD, pre-bronchodilator; SD, standard deviation.

**Table S5.** Oral corticosteroids (OCS) reduction in OCS-dependent patients with or without concomitant CRSwNP receiving benralizumab treatment, at 1-year of follow-up.

|  | **Patients with comorbid CRSwNP** | | **Patients with no comorbid CRSwNP** | |
| --- | --- | --- | --- | --- |
|  | **Baseline** | **1-year FUP** | **Baseline** | **1-year FUP** |
| Corticosteroids (CS)-dependant patients, n/N (%) | 17/68 (25.0%) | 5/72 (6.9%) | 36/114 (31.6%) | 19/128 (14.8%) |
| *OCS daily dose (mg), mean (SD)* | 21.7 (18.9) | 2.7 (4.8) | 18.7 (14.2) | 7.3 (10.7) |
| *OCS daily dose (mg), median [Q1-Q3]* | 10.0 [5.0-32.0] | 0.0 [0.0-5.0] | 15.0 [10.0-27.7] | 3.5 [0.0-10.0] |
| **Change in OCS use** |  |  |  |  |
| Patients achieving an OCS dose reduction ≥50%, n/N (%) | - | 12/17 (70.6%) | - | 21/36 (58.3%) |
| Patients achieving total OCS withdrawal, n/N (%) | **-** | 12/17 (70.6%) |  | 17/36 (47.2%) |
| Percentage reduction in mean OCS daily dose | **-** | 87.6% |  | 61.0% |
| Percentage reduction in median OCS daily dose | - | 100.0% |  | 76.7% |

Due to the nature of this real-world study, sample sizes vary due to missing, unavailable and/or not valid data. “N” represents the total number of patients with valid data.

Corticoid-dependent patients were defined as those who received maintenance systemic corticosteroids treatments during at least 3 months, within the 12 months prior to the index date.

CRSwNP, chronic rhinosinusitis with nasal polyposis; CS, corticosteroids; FUP, follow up; quartiles 1 and 3 [Q1-Q3]; OCS, oral corticosteroids; SD, standard deviation.


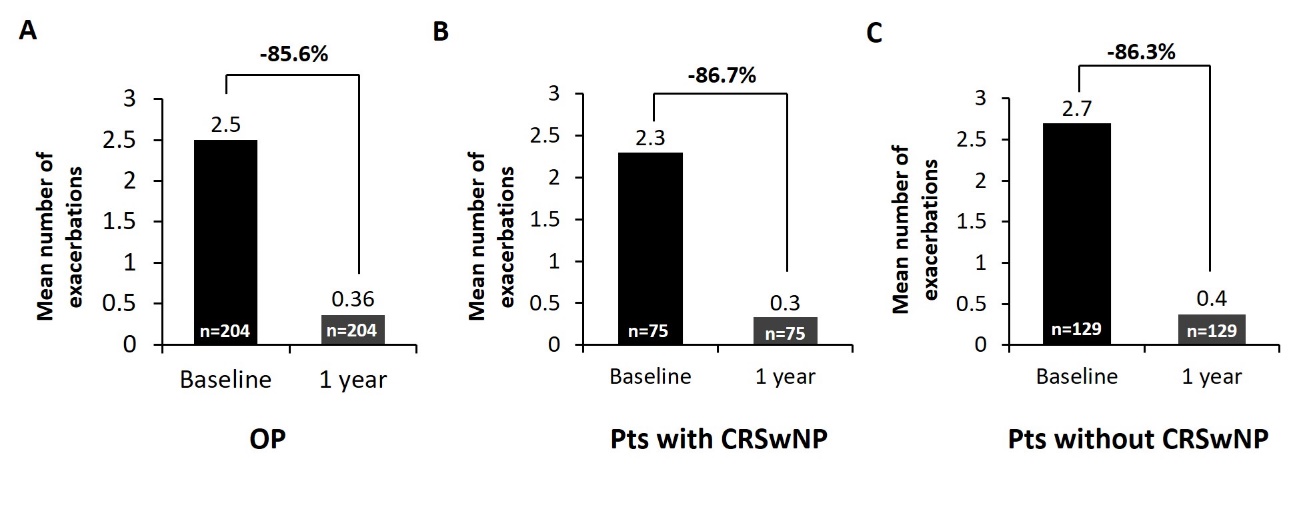
**Figure S1.** Change in the mean number of severe exacerbations from baseline to 1 year of follow-up in the OP (A) and in the subgroups of patients with (B) and without (C) comorbid CRSwNP. CRSwNP, chronic rhinosinusitis with nasal polyposis; OP, overall population; Pts, patients.


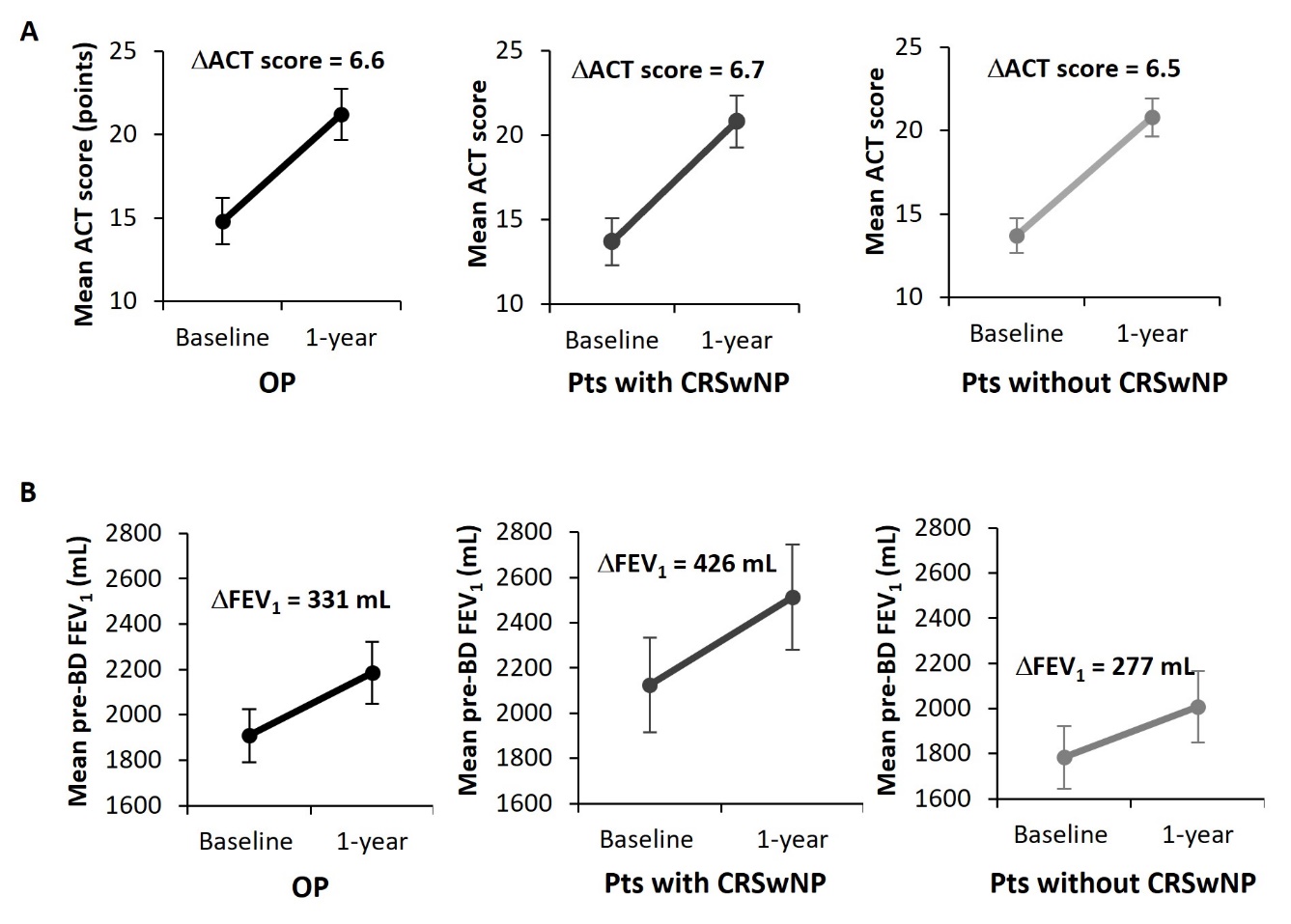
**Figure S2.** Improvements in asthma control and lung function in the OP and in the subgroups of patients with and without comorbid CRSwNP. (A) Change in the mean ACT score from baseline to 1 year of follow-up. (B) Change in the mean pre-BD FEV1 (mL) from baseline to 1-year of follow-up. Bars represent plus or minus one standard error of the mean. ACT, asthma control test; BD, bronchodilator; CRSwNP, chronic rhinosinusitis with nasal polyposis; FEV1, forced expiratory volume in 1 second; OP, overall population; Pts, patients.


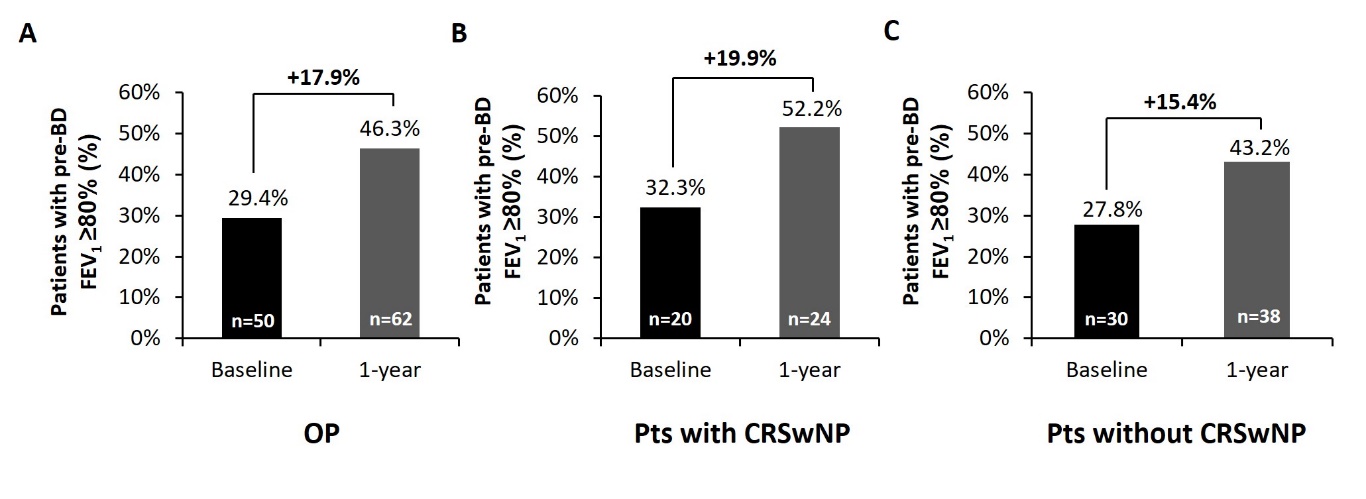
**Figure S3.** Proportion of patients with pre-BD FEV1 ≥80% of predicted at baseline and at 1 year of follow-up in the OP (A) and in patients with (B) and without comorbid CRSwNP (C). Percentages are calculated over the total number of patients with valid data. BD, bronchodilator; CRSwNP, chronic rhinosinusitis with nasal polyposis; FEV1, forced expiratory volume in 1 second; OP, overall population; Pts, patients.

**Figure S4.** UpSet plot showing the number and proportion of patients who met one or several of the pre-defined response criteria to benralizumab. Each column represents the percentage of patients who met one or several pre-defined criteria after 1 year of follow-up. Percentages are calculated over the total number of patients with valid data. Response criteria represented include the absence of severe exacerbations, asthma symptom control as defined by an ACT score ≥20, clinically relevant improvements in asthma control (as defined by an increase in the ACT score ≥3 points) and lung function (as defined by a pre-BD FEV1 ≥80% of predicted or a pre-BD FEV1 increase ≥100 mL) and no use of maintenance OCS. “N” represents the total of patients with available data. ACT, asthma control test; BD, bronchodilator; FEV1 forced expiratory volume in 1 second; OCS, oral corticosteroids.


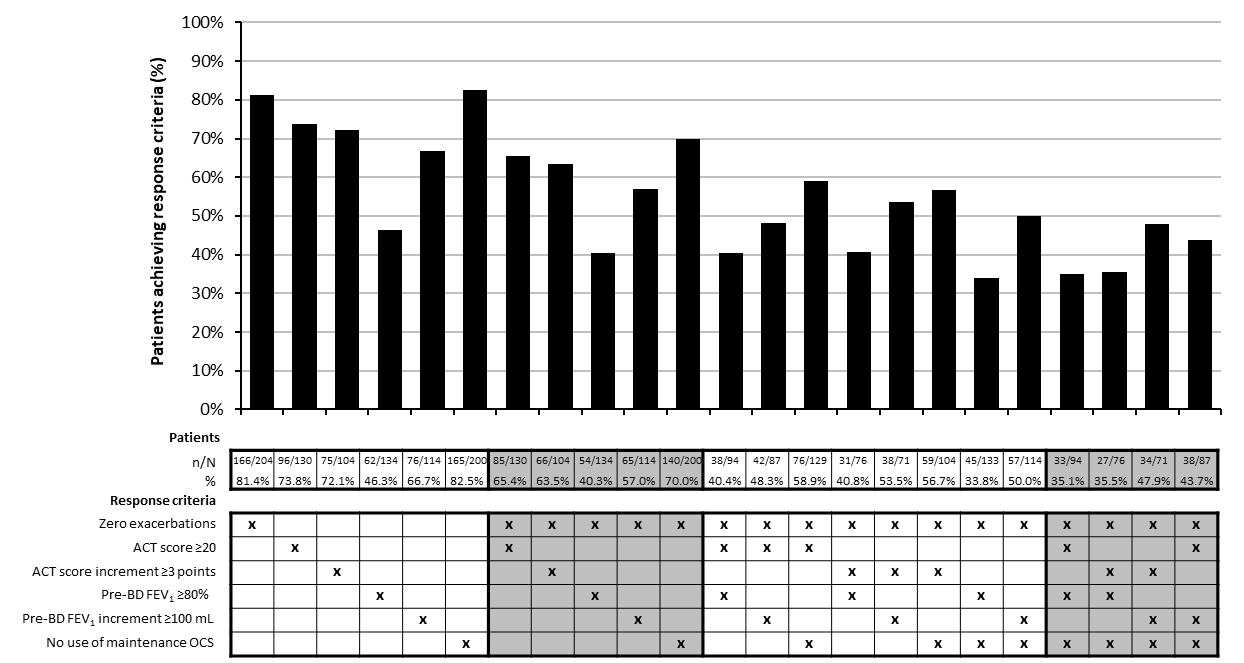


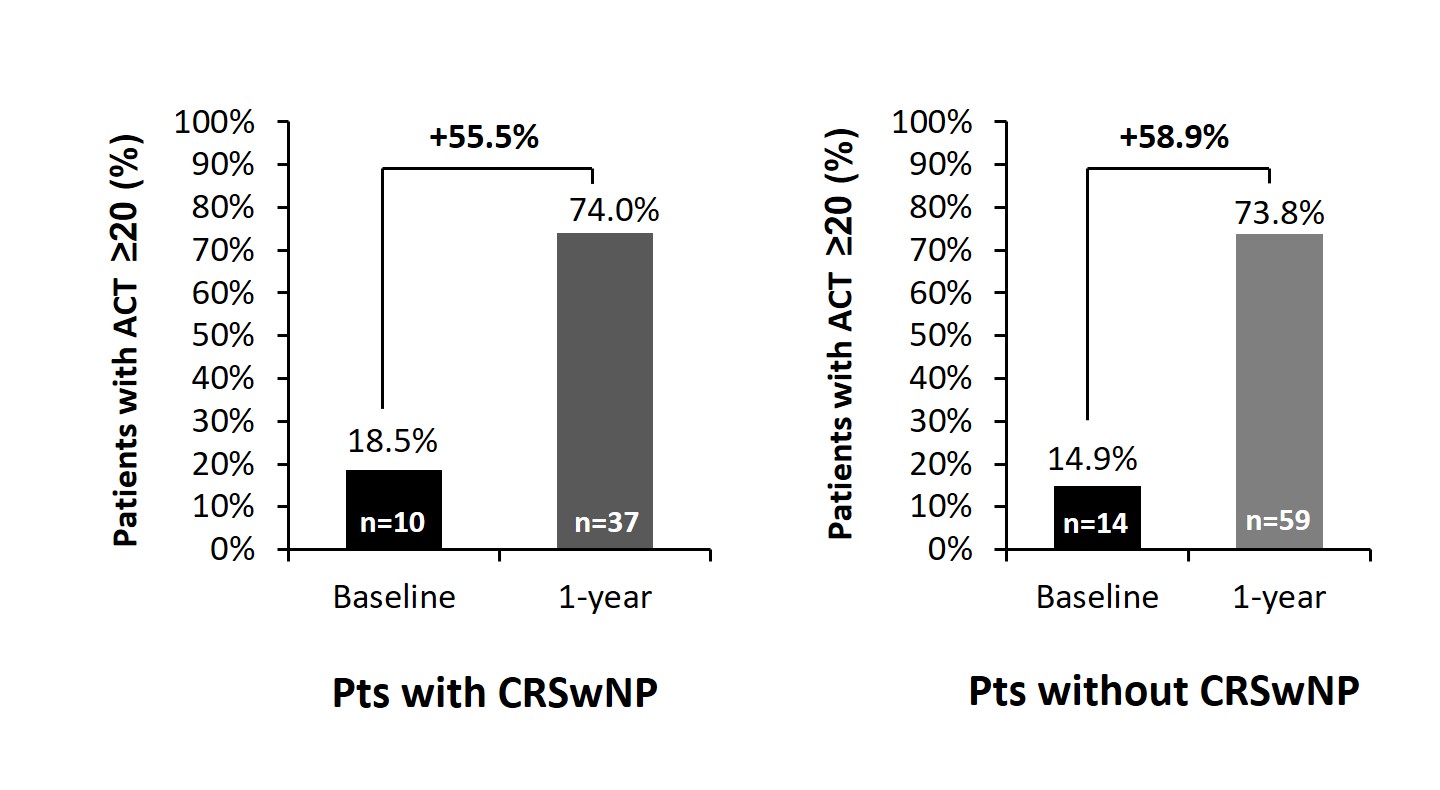
**Figure S5.** Proportion of patients with an ACT score ≥20 points at baseline and at 1 year of follow-up according to the presence or not of comorbid CRSwNP. Percentages are calculated over the total number of patients with valid data. ACT, asthma control test; CRSwNP, chronic rhinosinusitis with nasal polyposis; Pts, patients.


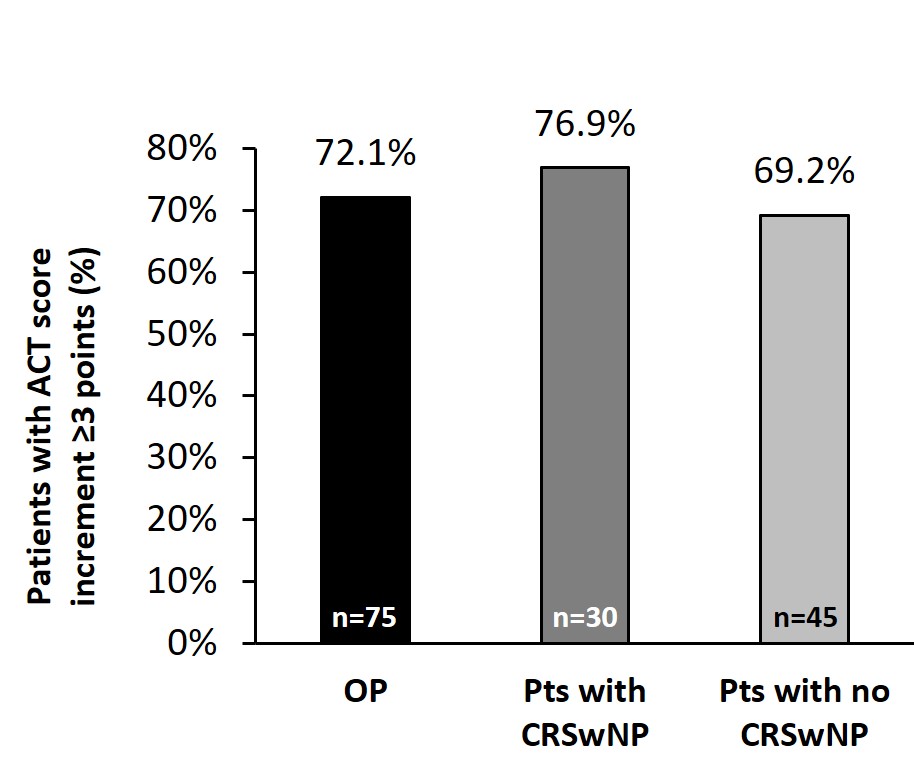
**Figure S6****.** Proportion of patients with an increase in the ACT score ≥3 points at 1 year of follow-up in the OP and in patients with and without comorbid CRSwNP. Percentages are calculated over the total number of patients with valid data. ACT, asthma control test; CRSwNP, chronic rhinosinusitis with nasal polyposis; Pts, patients.

**Figure S7.** Change in use of maintenance oral corticosteroids (OCS) over time in OCS-dependent patients with comorbid CRSwNP (n = 17). (A) Mean OCS dose reduction at 1 year of follow-up. (B) Estimated mean cumulative OCS exposure over 1 year of follow-up for patients continuing on study-entry mean OCS dose compared with patients treated with benralizumab and tapering the OCS dose. FUP, follow up; OCS, oral corticosteroids.


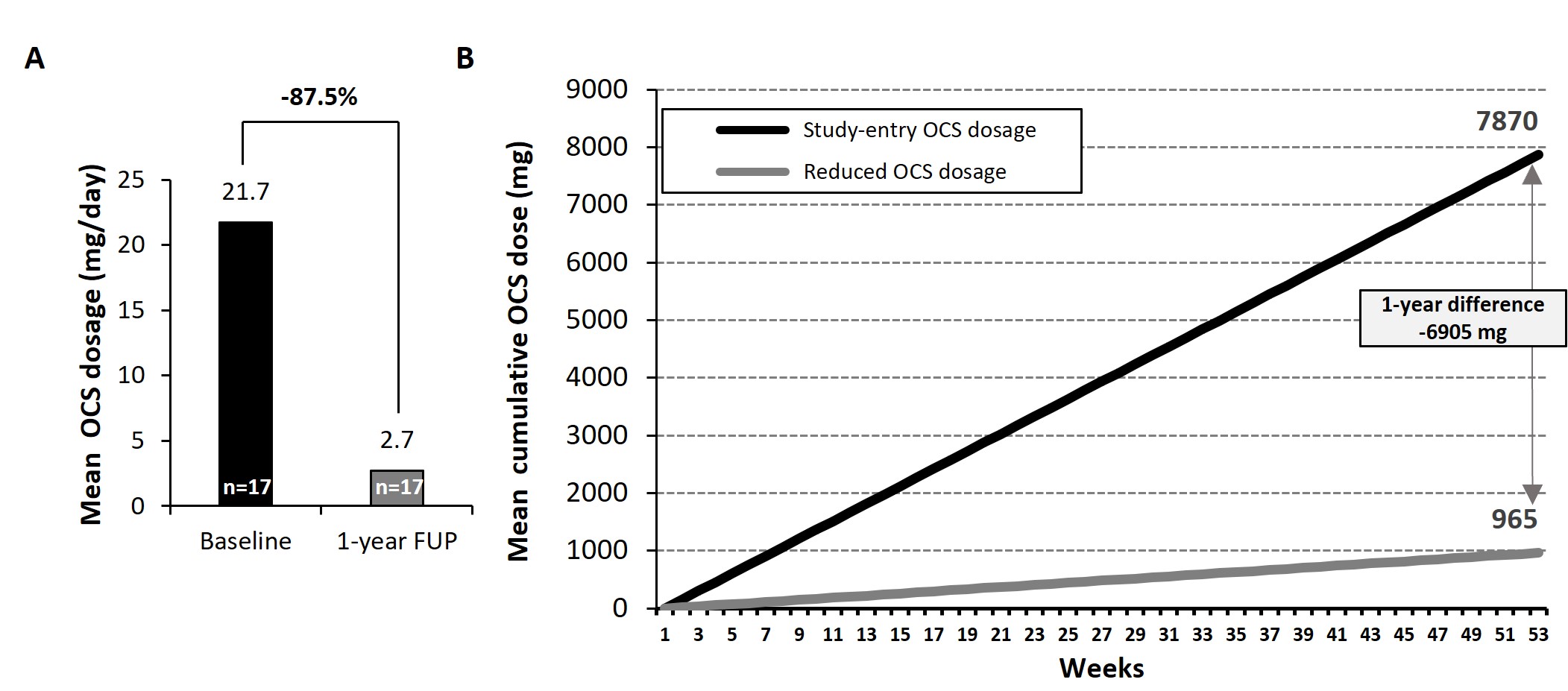

Supplement: Supplementary file 1 — Supplementary Material 1 [file 12931_2023_2539_MOESM1_ESM.docx]
